# Supplementary material for: Repeatability of evolution and genomic predictions of temperature adaptation in seed beetles
Source: Nat Ecol Evol. 2025 May 16;9(6):1061–74. doi: 10.1038/s41559-025-02716-5 (PMC12148939; doi:10.1038/s41559-025-02716-5)
Supplement: Supplementary file 1 — Supplementary Tables 1–6, Supplementary Figs. 1–11 and Supplementary Discussion: ‘Strength of selection on protein stability and epistasis for fitness at hot temperatures’. [file 41559_2025_2716_MOESM1_ESM.pdf]

nature ecology & evolution

Supplementary information https://doi.org/10.1038/s41559-025-02716-5

Repeatability of evolution and genomic predictions of temperature adaptation in seed beetles

In the format provided by the authors and unedited

Supplemental Material, Figures, and Tables

Phenotypic

Angle 23°C	Brazil	California	Yemen
Within	NA*	47.04	28.27
Between	66.94	67.86	72.28
Angle 35°C	Brazil	California	Yemen
Within	15.63	8.46	36.43
Between	25.07	35.10	53.63
Divergence 23°C	Brazil	California	Yemen
Within	0.00*	0.00*	0.00*
Between	0.02	-0.26	-0.28
Divergence 35°C	Brazil	California	Yemen
Within	0.24	0.00*	0.69
Between	0.16	0.21	0.21

Supplementary Table 1: Mean phenotypic angles (θ) and divergence ($E_d - S_d$) between lines of the same (within) and different (between) genetic background, for each selection regime. Within-background comparisons are represented by a single value (between the two replicates per origin). Asterisks represent estimates with high measurement error relative to vector length.

	Ne	# Putative Sites	# Putative Genes	$\overline{\lvert\Delta AF\rvert}$	$\frac{\overline{\lvert\Delta AF\rvert}}{gen}$	$\overline{\lvert S\rvert}$
Yemen1 (23°C)	244.29	61,458	2,405	0.546	0.009	0.0515
Yemen2 (23°C)	291.21	56,378	2,170	0.511	0.008	0.0490
Cali3 (23°C)	291.33	86,561	2,805	0.484	0.008	0.0440
Cali4 (23°C)	316.67	60,384	2,481	0.501	0.008	0.0374
Brazil5 (23°C)	194.23	34,216	1,490	0.607	0.010	0.0495
Brazil6 (23°C)	215.69	41,629	1,784	0.592	0.010	0.0448
Yemen7 (35°C)	178.38	32,804	1,343	0.663	0.010	0.0540
Yemen8 (35°C)	106.44	21,716	774	0.826	0.014	0.0660
Cali9 (35°C)	189.95	42,695	1,717	0.618	0.010	0.0552
Cali10 (35°C)	193.84	43,445	1,731	0.610	0.010	0.0480
Brazil11 (35°C)	161.86	28,758	1,217	0.658	0.010	0.0602
Brazil12 (35°C)	184.51	21,551	1,146	0.706	0.011	0.0596

Supplementary Table 2: Estimated Ne, number of putatively selected SNPs, number of putatively selected genes (genes with SNPs falling within protein coding regions), and the mean magnitude of allele frequency change, allele frequency change per generation, and selection coefficient per population. The magnitudes of allele frequency change and selection coefficient estimates are comprised of the putatively selected SNPs identified for each respective line.

	r
Yemen1 (23°c)	0.47
Yemen2 (23°c)	0.46
Cali.3 (23°c)	0.47
Cali.4 (23°c)	0.49
Brazil5 (23°c)	0.54
Brazil6 (23°c)	0.54
Yemen7 (35°c)	0.47
Yemen8 (35°c)	0.59
Cali.9 (35°c)	0.48
Cali.10 (35°c)	0.50
Brazil11 (35°c)	0.53
Brazil12 (35°c)	0.52

Supplementary Table 3: Estimated Pearson's correlation (r), between allele frequency change and estimated selection coefficient.

**Genomic**			
Angle 23°C	Brazil	California	Yemen
Within	65.77	80.10	73.00
Between	89.14	87.96	84.5
Angle 35°C	Brazil	California	Yemen
Within	71.10	73.98	71.75
Between	92.70	91.39	86.80
Divergence 23°C	Brazil	California	Yemen
Within	0.226	0.190	0.258
Between	-0.149	-0.122	-0.122
Divergence 35°C	Brazil	California	Yemen
Within	0.338	0.242	0.365
Between	0.014	-0.049	0.008

Supplementary Table 4: Mean genomic angles (θ) and divergence ($E_d - S_d$) between lines of the same (within) and different (between) genetic background, for each selection regime. Within-background comparisons are represented by a single value (between the two replicates per origin).

GO.ID 23°C	Term	Ann.	Sig.	Exp.	Adj. p-value
GO:0035556	intracellular signal...	195	13	6.73	0.0035
GO:0007156	homophilic cell adhe...	22	4	0.76	0.0062
GO:0035023	regulation of Rho pr...	29	4	1.00	0.0166
GO:0007205	protein kinase C-act...	8	2	0.28	0.0289
GO:0032940	secretion by cell	12	2	0.41	0.0344
GO:0007165	signal transduction	473	32	16.34	0.0410
GO:0007186	G protein-coupled re...	148	11	5.11	0.0487
GO:0040007	growth	20	8	0.69	1.5e-07
GO.ID 35°C	Term	Ann.	Sig.	Exp.	Adj. p-value
GO:0007156	homophilic cell adhe...	22	2	0.12	0.006
GO:0007018	microtubule-based mo...	46	2	0.25	0.025
GO:0019319	hexose biosynthetic ...	5	1	0.03	0.027
GO:0006013	mannose metabolic pr...	5	1	0.03	0.027

Supplementary Table 5: Gene set enrichment analysis for genic targets of selection shared across all 6 cold-adapted replicates (top), and all 6 hot-adapted replicates (bottom).

Shared Genomic

Angle 23°C	Yemen	California	Brazil
Within	74.56	75.29	71.67
Between	81.87	83.45	84.03
Angle 35°C	Yemen	California	Brazil
Within	70.52	75.07	69.19
Between	84.69	89.15	90.49
Divergence 23°C	Yemen	California	Brazil
Within	45.53	34.07	36.98
Between	3.9	8.25	7.6
Divergence 35°C	Yemen	California	Brazil
Within	60.36	44.34	57.08
Between	22.39	17.12	18.63

Supplementary Table 6: Mean genomic angles (θ) and divergence ($E_d - S_d$) of within- and between-background comparisons for each selection regime using selected sites which were variable in the ancestors of all populations. Within-comparisons are represented by a single value between the two replicates per origin.

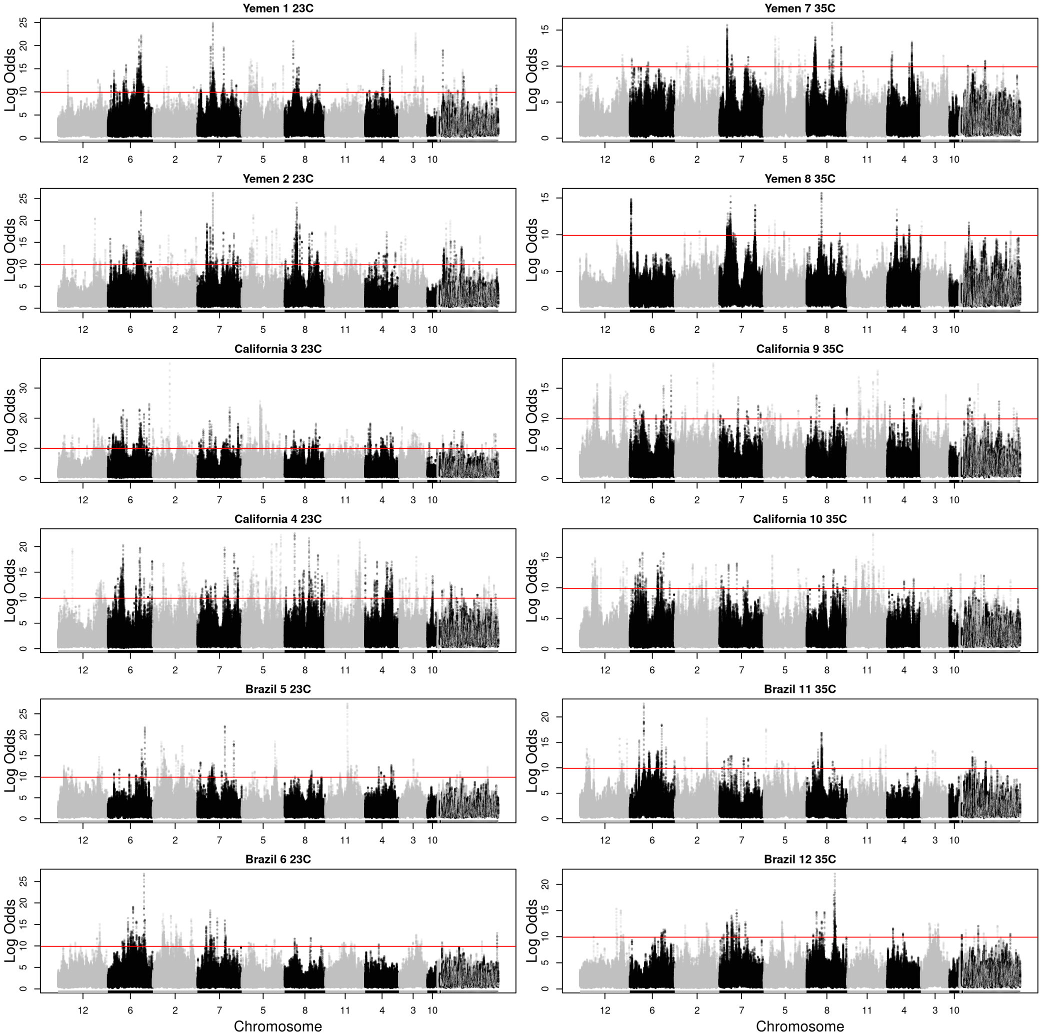

Supplementary Figure 1: **Manhattan plots of rolling average log odds p-values of allele frequency change across the genome.** Rolling average (window size = 20 SNPs) p-values are shown for each line across all selected sites using `R` package `RcppRoll` (v.0.3.0). The largest 10 scaffolds (i.e., chromosomes) are named. The significance threshold of log odds ($log(\frac{0.99995}{0.00005})$) is given by the horizontal red line.

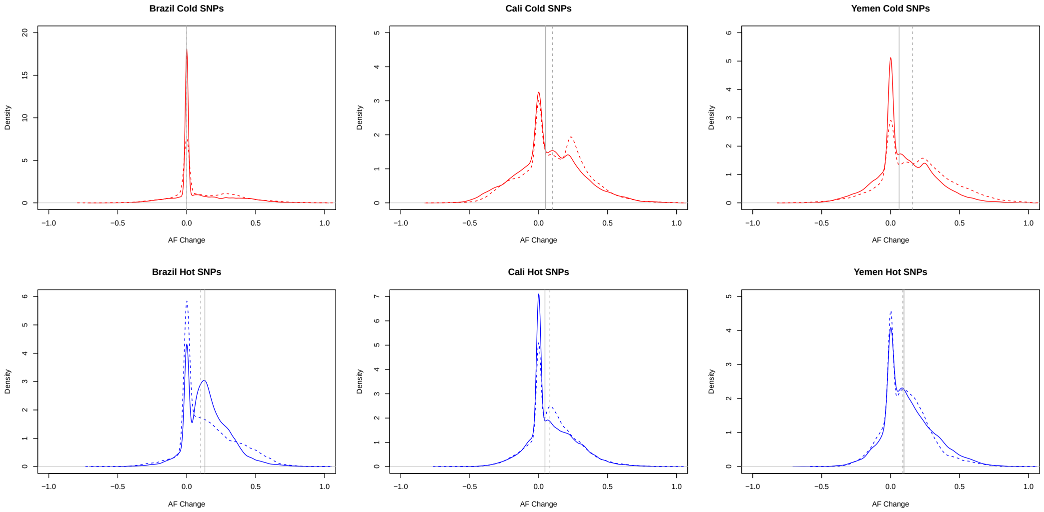

Supplementary Figure 2: **Genome-wide Manhattan plots of the rolling average log odds of allele frequency change.** Allele frequency changes in hot (red) and cold (blue) lines for candidate SNPs identified in the alternative thermal regime. For each selected SNP in a thermal regime, we quantified allele frequency changes in the alternative thermal regime (i.e., cold-associated SNPs in heat-adapted lines, and hot-associated SNPs in cold-adapted lines). Allele frequency changes were calculated such that positive values indicate a change in the same direction as found in the thermal regime where the SNP was identified as under putative selection (implying synergistic effects on fitness across temperatures), whereas negative values indicate a change in the opposite direction (implying antagonistic effects on fitness across temperatures). The two replicate lines per background are shown separately as solid and dashed colored lines. Vertical solid and dashed grey lines indicate the median allele frequency change of the respective replicate line. While there are many examples of allele frequency changes going in both the same and opposite direction in the two thermal regimes, there is a large over-representation of selected SNPs showing no change in the alternative thermal regime, and the mean change even seems to be slightly positive. This suggests that SNPs involved in adaptation during the experiment tended to have effects that were private to each temperature and provides little evidence for a dominating role of antagonistic pleiotropy

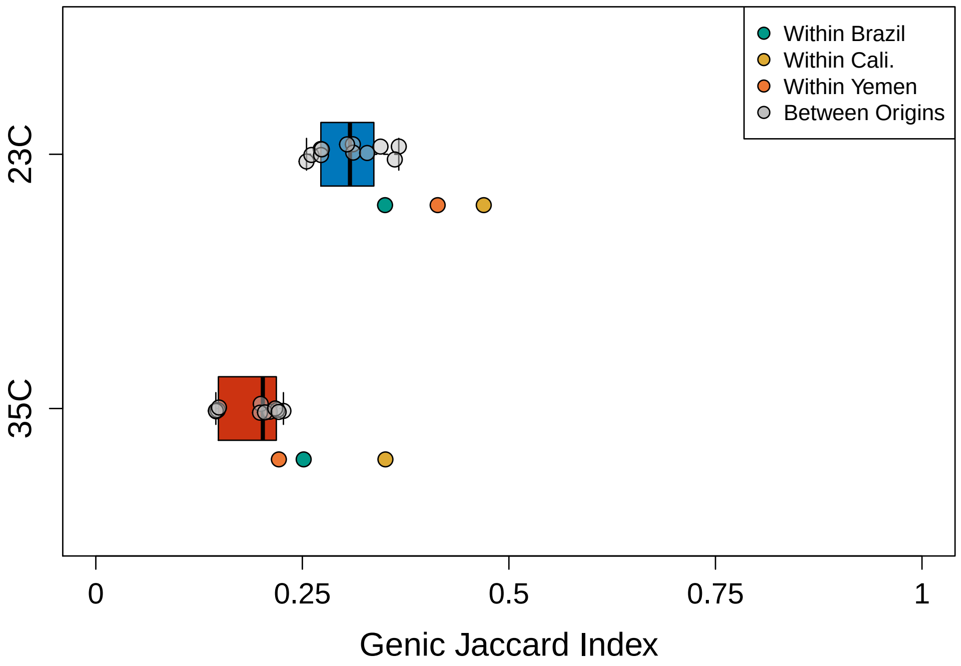

Supplementary Figure 3: **Distribution of Jaccard indices for genic targets of selections between lines within temperature regimes.** Distributions of angles and divergences are separated by between-origin (grey points) within-origin (colored points) pairwise comparisons. Between-origin boxplots display the median (solid center line), interquartile range (bounds of the box), and range (whiskers) of the data. A Jaccard index of 0 indicates no overlap in genic targets, while an index of 1 indicates complete overlap in genic targets.

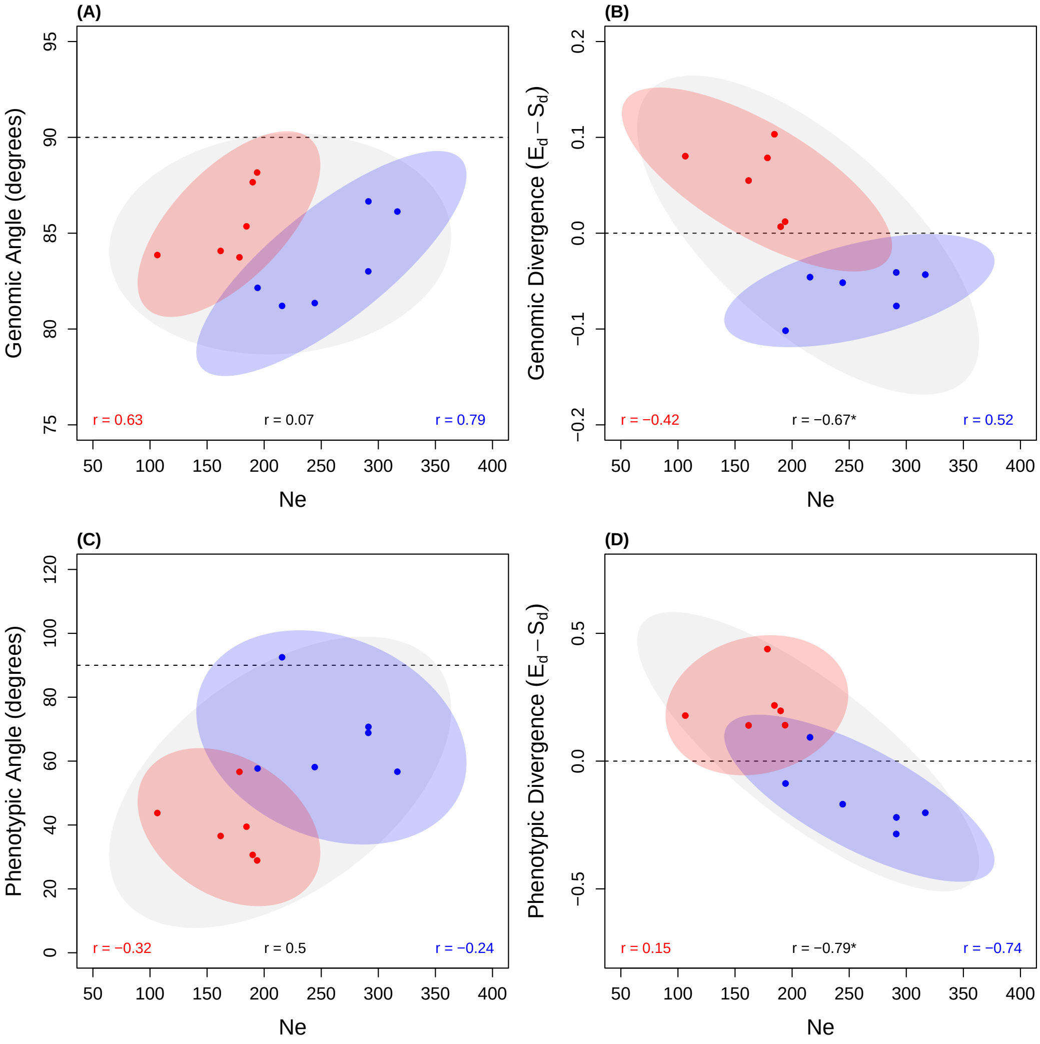

Supplementary Figure 4: **Correspondence between mean measures of phenotypic (A–B) and genomic (C–D) repeatability, and estimates of effective population size (*Ne*) per line.** In each plot, points are colored by regime. Confidence ellipses (95%) and correlations are given both per regime (red, 35°C; blue, 23°C) and across regimes (grey) based on line means. Significant correlations are designated with an asterisk.

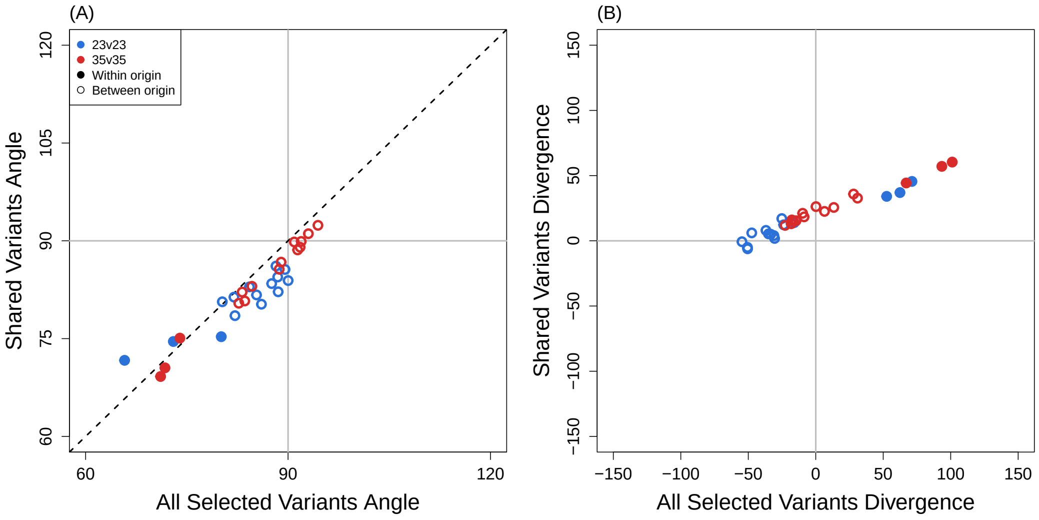

Supplementary Figure 5: **Comparison of repeatability measures between different sets of SNPs.** SNPs were included by either being selected in any one population or by both being selected in any one population and segregating in all populations. The black dashed line represents the identity line.

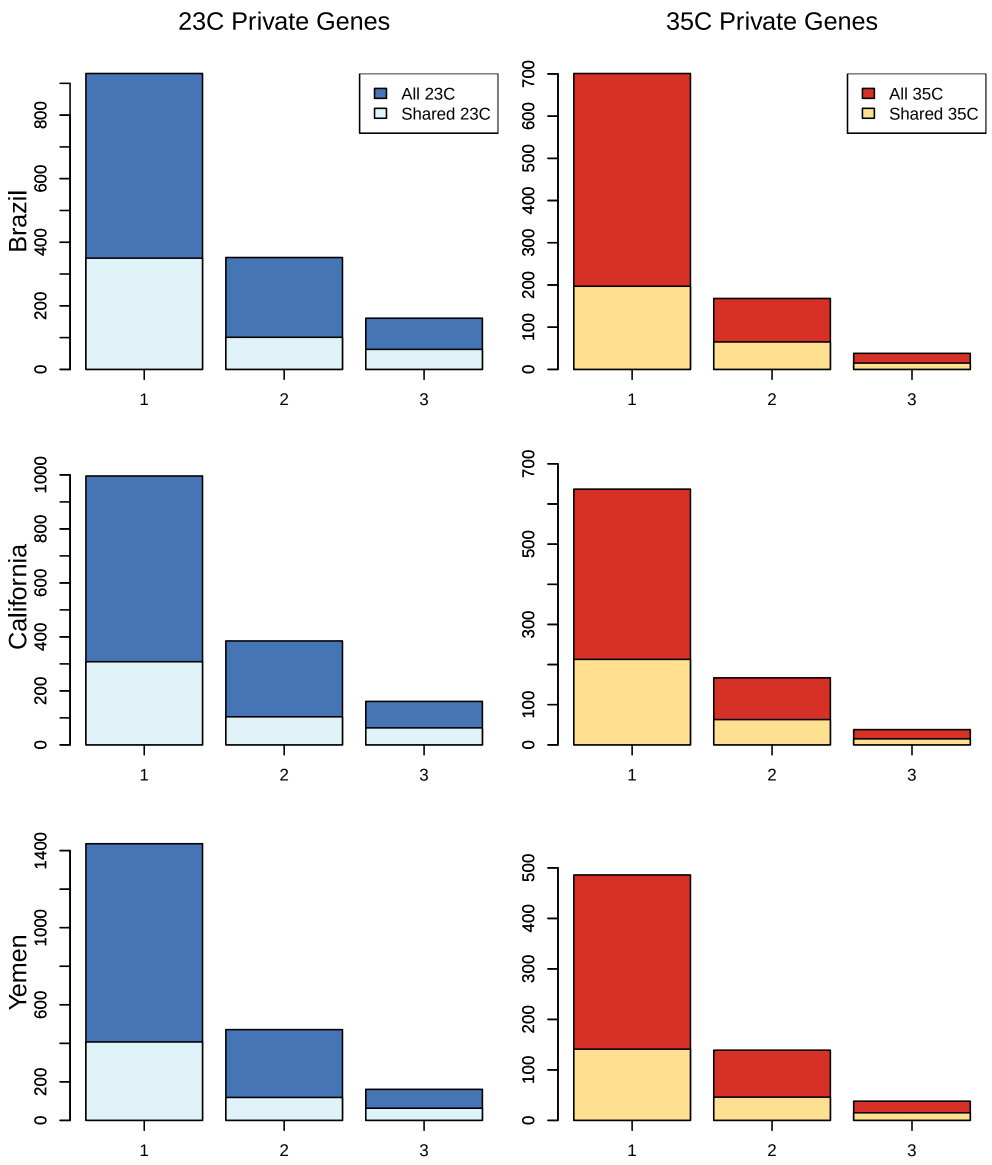

Supplementary Figure 6: **Distributions of the number of privately selected genes shared between populations for each thermal regime.** Genes were found to be under selection in either a single (1) or (2) backgrounds, or on all three backgrounds (3). The distributions for both the entire set of selected sites (n=475,194) and sites which are polymorphic across all ancestral backgrounds (n=119,630) are shown in different shades. Chi-square tests show no significant differences between the distributions for all selected sites versus shared sites for any of the three comparisons ($p > 0.05$).

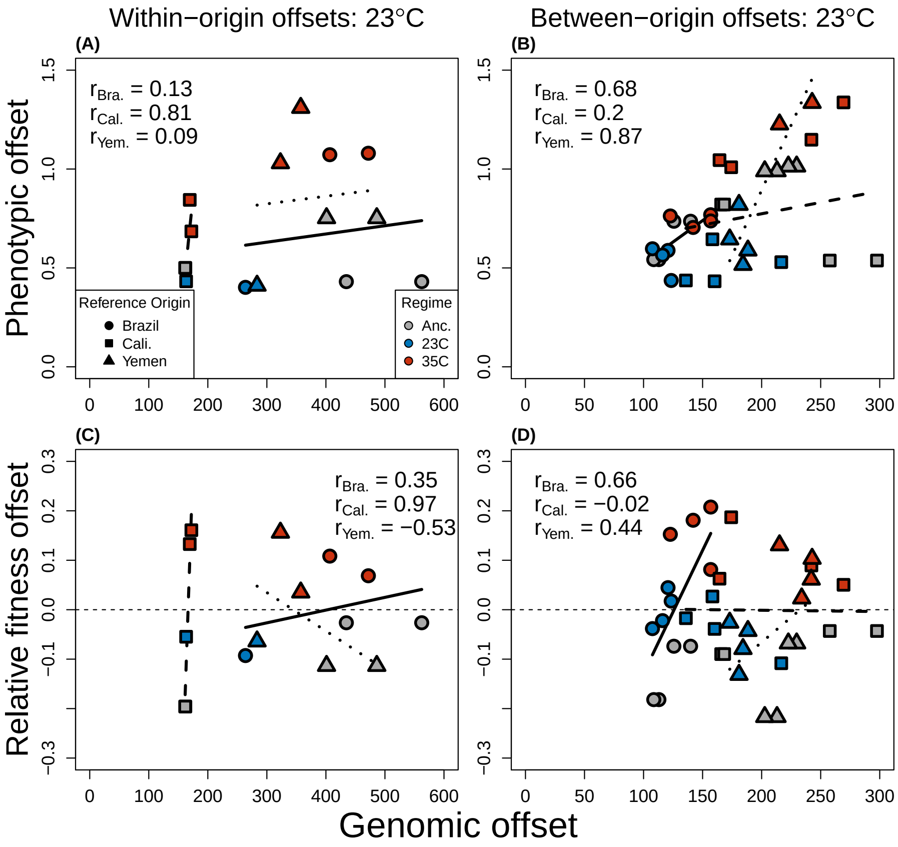

Supplementary Figure 7: **Genomic predictors of phenotypic divergence and relative fitness offsets at cold temperature**. Genomic offsets were calculated per reference origin based on SNPs whose p-values for allele frequency change fell within the top 0.001th quantile (n=10546–10887). Phenotypic offsets were calculated as the Euclidean distance in scaled trait-space between the tested line and the reference line. Relative fitness offsets were calculated as the laboratory fitness ($\frac{LRS}{Dev.Time}$) of the tested line relative to that of the reference line. Offsets are organized by within-origin comparisons (A,C) and between-origin comparisons (B,D). Correlations and regression lines are colored by the geographic origin of the reference line.

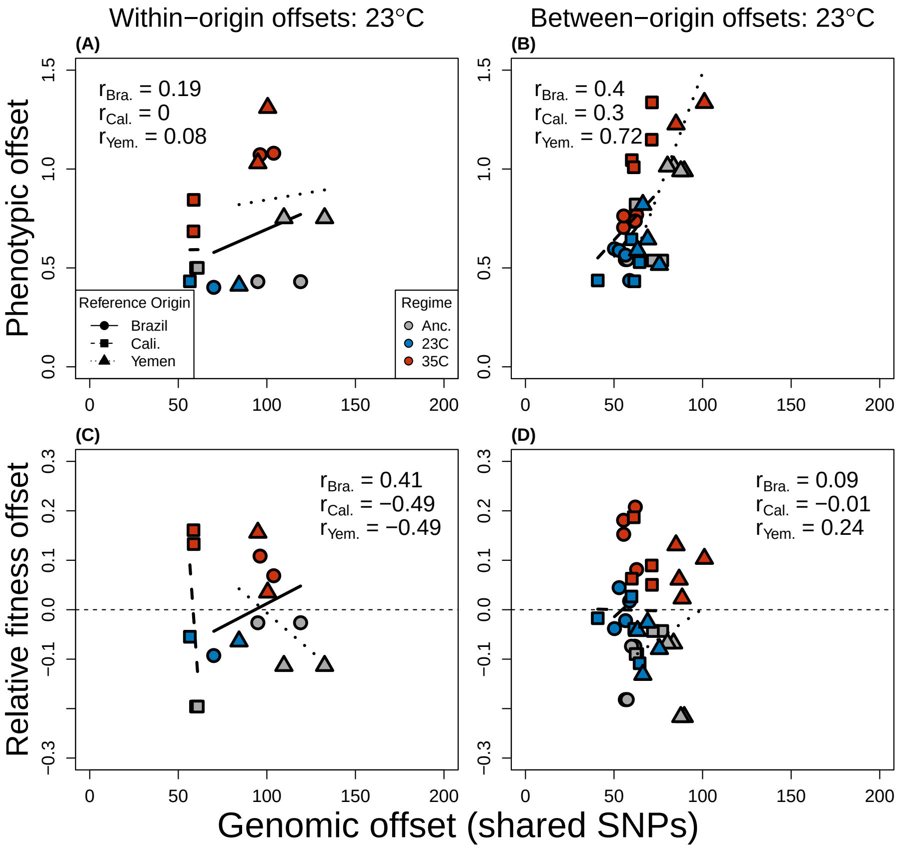

Supplementary Figure 8: **Genomic predictors of phenotypic divergence and relative fitness offsets at cold temperature**. Genomic offsets were calculated per reference origin based on SNPs which were polymorphic among all ancestors and whose p-values for allele frequency change fell within the top 0.001th quantile (n=2750–2804). Phenotypic offsets were calculated as the Euclidean distance in scaled trait-space between the tested line and the reference line. Relative fitness offsets were calculated as the laboratory fitness ($\frac{LRS}{Dev.Time}$) of the tested line relative to that of the reference line. Offsets are organized by within-origin comparisons (A,C) and between-origin comparisons (B,D). Correlations and regression lines are colored by the geographic origin of the reference line.

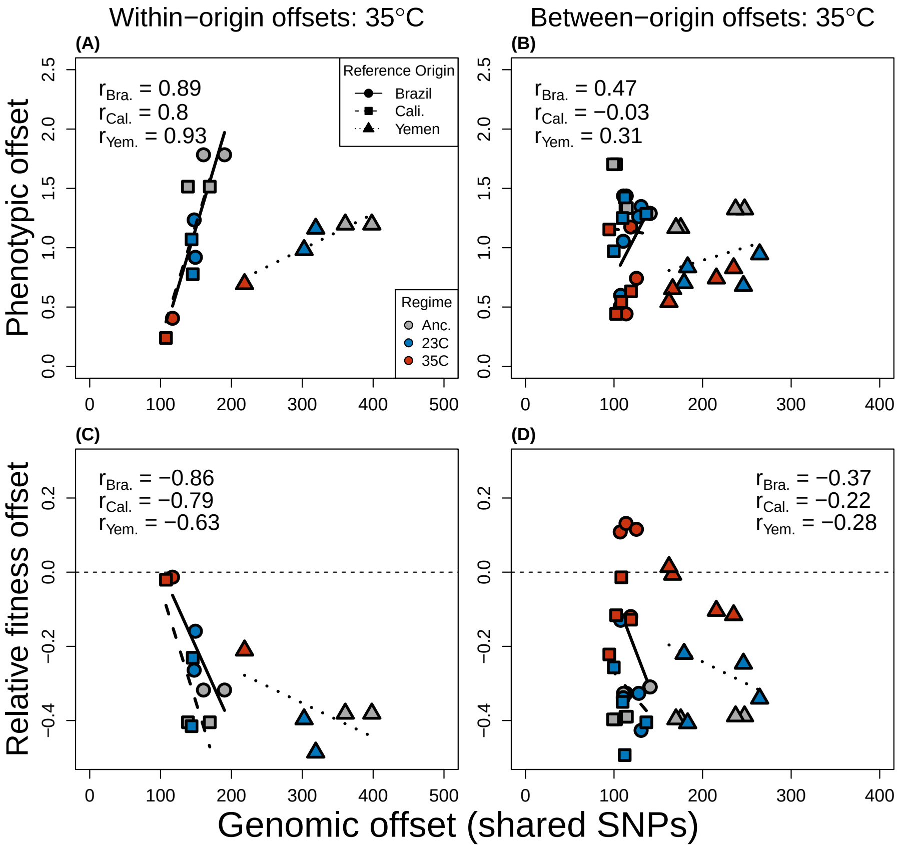

Supplementary Figure 9: **Genomic predictors of phenotypic divergence and relative fitness offsets at hot temperature using shared polymorphisms**. Genomic offsets were calculated per reference origin based on SNPs which were polymorphic among all ancestors and whose p-values for allele frequency change fell within the top 0.001th quantile (n=2750–2787). Phenotypic offsets were calculated as the Euclidean distance in scaled trait-space between the tested line and the reference line. Relative fitness offsets were calculated as the laboratory fitness ($\frac{LRS}{Dev.Time}$) of the tested line relative to that of the reference line. Offsets are organized by within-origin comparisons (A,C) and between-origin comparisons (B,D). Correlations and regression lines are colored by the geographic origin of the reference line.

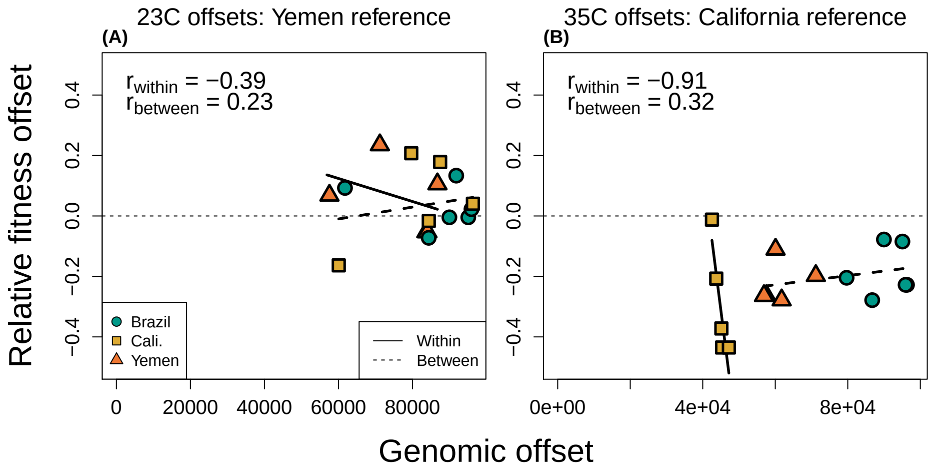

Supplementary Figure 10: **Genomic predictors of relative fitness offsets using lines with the greatest performance per thermal regime as the reference.** Genomic offsets were calculated based on all putatively selected SNPs identified in any line replicate, for each thermal regime separately (Yemen at 23°C, California at 35°C). Allele frequency changes were not scaled by selection coefficients as for other genomic offsets. Relative fitness offsets were calculated as the tested line's laboratory fitness ($\frac{LRS}{Dev.Time}$) relative to that of the reference line. Correlations and regression slopes are separated by predictions within and between origins.

A hypothesis for how temperature extremes affect epistasis for fitness based on the thermodynamics of enzyme performance.

Biological rates of ectotherms show an empirically well-described, roughly exponential, increase with temperature that closely mirrors the thermodynamic properties of enzyme reactions [1, 2]. This pattern occurs because biological rates are governed at the molecular level by the catalytic reaction rate, k_{cat}. Based on transition state theory [3, 4]:

$$k_{cat} = k_0 e^{-\Delta G^a/RT} \tag{1}$$

where ΔG^a is the Gibbs free energy of activation required for the enzymatic reaction to occur (kcal mol^{-1}), R is the universal gas constant (0.002 kcal mol^{-1}), T is temperature measured in Kelvin, and $k_0 = \kappa k_B T/h$ where κ is a rate- and species-specific constant, k_B is the Boltzmann constant, and h is Planck's constant. ΔG^a is comprised by an enthalpy term (ΔH^a) and a temperature-dependent entropy term (ΔS^a):

$$\Delta G^a(T) = \Delta H^a - T\Delta S^a \tag{2}$$

Warm temperature increases the entropy term ($\Delta S^a T$), reducing ΔG^a. Equations S1 and S2 thus describe an exponential increase in reaction rate with temperature.

Strength of selection on catalytic rates and epistasis for fitness at cold temperatures

To form a hypothesis for how natural selection acts on catalytic rates at different temperatures, we incorporate ecological realism into predictions by assuming that the temperature-driven exponential increase in reaction rates will not have a 1:1 mapping with reproductive output, due to constraints on reproductive output set by other factors than temperature. At some point, suboptimal co-expression of correlated traits (evolutionary constraints), and/or limited nutrients or reproductive opportunities (ecological constraints) should make fitness pay-offs follow a pattern of diminishing returns with increases in catalytic rates [5–7] until temperature-dependent

physiological rates are no longer the limiting factor on reproductive output (Fig. S1A). We describe these dynamics by assuming that reproductive rates are a logistic function of temperature, where the initial exponential increase in reproductive rate is explained by temperature-driven increases in catalytic rates and a release from thermodynamic constraints as temperatures warm, but where diminishing returns result from ecological constraints at higher temperatures:

$$R_B = \frac{B_{max}}{1 + \frac{(B_{max} - B_{T_{ref}})}{B_{T_{ref}}} \times e^{\frac{-\Delta S(T - Tref)}{RT}}} \tag{3}$$

where B_{max} is the maximum reproductive rate of the organism, and $B_{T_{ref}}$ is reproductive output as a function of ΔG^a at the reference temperature, T_{ref}. We introduce a beneficial mutation that increases reproductive rate at the reference temperature (i.e., $B_{T_{ref}}$) by 10% through effects on ΔH^a ($\Delta\Delta H^a = -0.055$ kcal/mol). We then estimate selection on this mutation across temperatures by comparing wildtype reproductive rate to that of the mutant (Fig. S1A, B). To estimate epistasis and the dependency of natural selection on genetic background, we performed these calculations on three genetic backgrounds with wildtype $B_{T_{ref}}$ set to 40, 50 or 60 at $T_{ref} = 23°C$, with $B_{max} = 100$ in all cases. We further set ΔS to 0.50 kcal/mol K^{-1} based on values from the literature [8, 9].

This scenario generates strong positive selection on the introduced mutation across all three backgrounds at cold temperature ($s = 0.1$), while selection is weaker at intermediate benign temperature ($s \approx 0.005$) and at hot temperature the mutation is effectively neutral ($s \approx 0$) (Fig. S1B). This effect arises because cold temperatures act rate-limiting on reproductive output on all three backgrounds to an extent that far exceeds any rate-limiting ecological process. Once ecological constraints come into play at warmer temperatures, selection on the mutation weakens, but to similar extent across backgrounds, and little epistasis results.

Strength of selection on protein stability and epistasis for fitness at hot temperatures

Organisms that experience temperatures that exceed their thermal optimum suffer from increased cellular stress and many physiological processes fail. This results in an exponential increase in molecular failure rates and cellular damage [1, 10]. One major component of cellular damage and decreased fitness at hot temperature is attributed to a reduction in the proportion of functional enzyme due to reversible inactivation via protein unfolding [11–15]. While this loss of protein stability is reversible, the reduction of properly folded protein leaves fewer molecules ready for work [14], and can cause excessive cellular toxicity as misfolded proteins clog up the cellular environment [16]. While several processes contribute to the observed exponential increase in death rates with temperature [1], here we use the example of protein folding stability to illustrate how thermodynamics predict temperature-dependent selection due to the rich empirical data allowing parameterization of the calculations. The proportion of folded enzyme ready to catalyse reactions follows a Boltzmann probability as a function of the Gibbs free energy of folding, ΔG^f, which is thus a measure of protein stability [12]

$$P_{fold} = \prod_{1}^{i} = \frac{1}{1 + e^{-\frac{\Delta G^f_{T_{ref}} - \Delta S f (T - T_{ref})}{RT}}} \qquad (4)$$

where stability (ΔG^f) is given for each of a set of i proteins that act in sequence and which effects are multiplicative [8, 14]. At a benign temperature of 25°C, most natural proteins occur in functional (properly folded) state, and mean $\Delta G^f \approx 7$ kcal mol^{-1} [12, 17]. Warm temperatures increase entropy $(\Delta S^f(T - T_{ref}))$, reducing ΔG^f, which leads to a rapid non-linear reduction in the fraction of functional enzyme (Fig. S1C). We introduce a beneficial mutation that increases folding stability at the reference temperature (T_{ref}) and estimate selection on this mutation across temperatures by comparing the fraction of properly folded enzyme of the wildtype to that of the mutant (Fig. S1C). For the effect size of the mutation, we chose to increase folding stability by decreasing ΔH^f by 1 kcal mol^{-1} ($\Delta\Delta H^f = -1.0$ kcal/mol) based on empirical estimates [9, 17, 18]. We did this on three

genetic backgrounds with the wildtype ΔH^f in the enzyme receiving a mutation equal to 7, 9, or 11 kcal mol^{-1} at $T_{ref} = 23$°C, which are typical stabilities observed across ectotherms (Dill et al. 2011). For other parameters we assumed $\Delta S = 0.50$ kcal mol^{-1} K^{-1} and $i = 100$ proteins (e.g. [8, 17]).

In this scenario, the mutation acts as effectively neutral on all backgrounds at cold temperature ($s \approx 0$). At intermediate temperature, there is selection on the background with the most unstable wildtype configuration ($s \approx 0.01$) whereas the mutation is still effectively neutral on the other two backgrounds. At hot temperature the mutation is under strong selection on the backgrounds with low ($s > 0.1$) and intermediate ($s \approx 0.03$) wildtype stability, whereas the mutation is still effectively neutral ($s \approx 0$) on the background with most stable wildtype configuration (Fig. S1D), signifying substantial epistasis for fitness. This effect arises because proteins evolve marginal stability up until a point where selection is too weak to increase stability further [14, 19]. Around this zone of marginal stability, new mutations with conditional effects that are weak and effectively neutral at colder temperatures can accumulate, which affects subsequent selection on new genetic variants.

Protein folding can also be compromised by acute cold stress [1, 20]. However, such cold temperatures are typically not experienced during the active seasons of tropical and sub-tropical insects [21, 22], as the case for *C. maculatus* [23], and were therefore not modeled. Moreover, stressful temperatures affect organisms in a multitude of ways [1, 2, 5]. Thus, our particular example using protein folding is merely meant to illustrate a general principle and was chosen based on the simple fact that the modeled relationships are empirically well-supported in qualitative sense (see above). Other physiological phenomena that scale with temperature, such as metabolic expenditure [24] and the production of harmful reactive oxygen species [25], are likely to simultaneously contribute to increasing molecular failure rates at stressful temperature. If such processes follow similar patterns with temperature (i.e. marginally robust at the thermal optimum and governed by the same thermodynamics principles), similar predictions for the strength of selection and epistasis for fitness would result.

Yet, we have only illustrated predictions based on general, and very

simplified, principles founded in thermodynamics. Our predictions assume multiplicative action between genes (or pathways) in effects on catalytic rates (Fig. S1A) and protein stability (Fig. S1C) that result in epistasis for fitness via non-linear (and temperature-dependent) mapping to fitness. We argue that these effects are general and may explain broad patterns in the average extent of epistasis for fitness across temperatures. However, at the more fine-grained scale, molecular epistasis is the result of intricate and interdependent molecular processes which are likely to cause epistatic interactions among genes governing interacting pathways, which are likely omnipresent at any temperature.

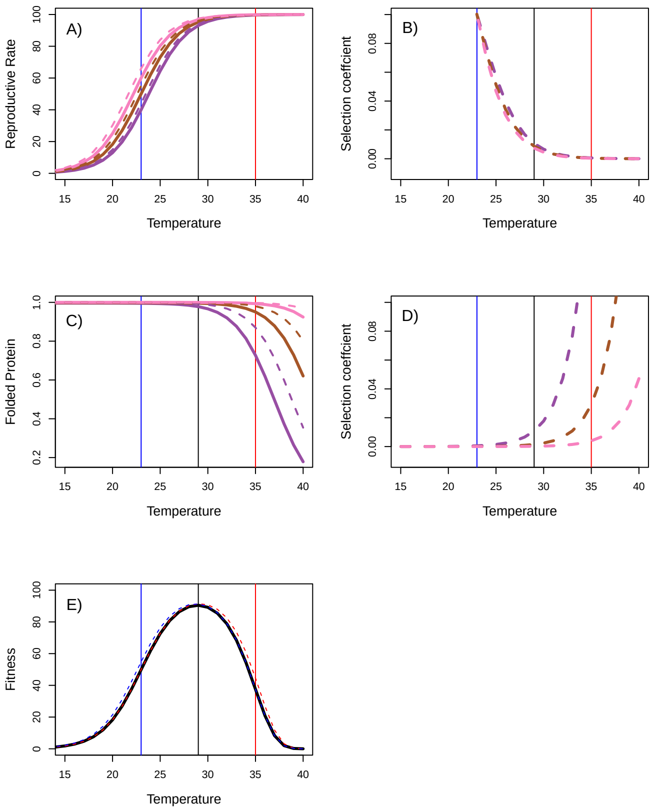

Supplementary Figure 11: **A hypothesis for temperature-dependent epistasis for fitness.** A) Reproductive rate increases exponentially with temperature at the colder range due to relaxation of thermodynamics constraints on reaction rates, but starts to follow a pattern of diminishing returns at warmer temperatures due to ecological constraints. Illustrated for three genetic backgrounds (pink, brown, and purple) with wildtypes (solid lines) and a mutant (broken lines) with a 10% increase in reproductive rate at 23°C. B) This results in strong selection on the mutation at cold temperature, but weak selection at hot temperature, and no epistasis. C) Warm temperatures increase molecular failure rates, for example via decreased protein stability. Illustrated for three genetic backgrounds with stable (pink), intermediate (brown) and unstable (purple) wildtype protein. A mutation increasing stability (broken lines) was introduced on each background. D) Selection on the mutation is weak at cold temperature for all backgrounds but can become under very strong selection at hot temperatures, depending on the wildtype protein stability, and strong epistasis for fitness results. E) Protein fitness as the product of reproductive rate and protein stability is depicted for the wildtype (black), and the mutants with increased reaction rate (blue) and increased protein stability (red), calculated for the yellow background.

Supplementary Literature Cited

1. Somero, G. N., Lockwood, B. L. & Tomanek, L. *Biochemical adaptation: Response to Environmental Challenges from Life's Origins to the Anthropocene* (Oxford university press, 2017).
2. Angilletta, M. J. *Thermal adaptation: a theoretical and empirical synthesis* (Oxford University Press, 2009).
3. Evans, M. G. & Polanyi, M. Some applications of the transition state method to the calculation of reaction velocities, especially in solution. *Transactions of the Faraday Society* **31,** 875–894 (1935).
4. Eyring, H. The activated complex in chemical reactions. *The Journal of Chemical Physics* **3,** 107–115 (1935).
5. Clarke, A. Is there a universal temperature dependence of metabolism? *Functional Ecology* **18,** 252–256 (2004).
6. Berger, D., Walters, R. & Gotthard, K. What limits insect fecundity? Body size-and temperature-dependent egg maturation and oviposition in a butterfly. *Functional Ecology* **22,** 523–529 (2008).
7. Dell, A. I., Pawar, S. & Savage, V. M. Systematic variation in the temperature dependence of physiological and ecological traits. *Proceedings of the National Academy of Sciences* **108,** 10591–10596 (2011).
8. Chen, P. & Shakhnovich, E. I. Thermal adaptation of viruses and bacteria. *Biophysical journal* **98,** 1109–1118 (2010).
9. Dill, K. A., Ghosh, K. & Schmit, J. D. Physical limits of cells and proteomes. *Proceedings of the National Academy of Sciences* **108,** 17876–17882 (2011).
10. Jørgensen, L. B., Ørsted, M., Malte, H., Wang, T. & Overgaard, J. Extreme escalation of heat failure rates in ectotherms with global warming. *Nature* **611,** 93–98 (2022).
11. Bloom, J. D. *et al.* Thermodynamic prediction of protein neutrality. *Proceedings of the National Academy of Sciences* **102,** 606–611 (2005).
12. DePristo, M. A., Weinreich, D. M. & Hartl, D. L. Missense meanderings in sequence space: a biophysical view of protein evolution. *Nature Reviews Genetics* **6,** 678–687 (2005).

13. Bershtein, S., Serohijos, A. W. & Shakhnovich, E. I. Bridging the physical scales in evolutionary biology: from protein sequence space to fitness of organisms and populations. *Current opinion in structural biology* **42,** 31–40 (2017).

14. Echave, J. & Wilke, C. O. Biophysical models of protein evolution: understanding the patterns of evolutionary sequence divergence. *Annual review of biophysics* **46,** 85–103 (2017).

15. Agozzino, L. & Dill, K. A. Protein evolution speed depends on its stability and abundance and on chaperone concentrations. *Proceedings of the National Academy of Sciences* **115,** 9092–9097 (2018).

16. Allan Drummond, D. & Wilke, C. O. The evolutionary consequences of erroneous protein synthesis. *Nature Reviews Genetics* **10,** 715–724 (2009).

17. Chen, P. & Shakhnovich, E. I. Lethal mutagenesis in viruses and bacteria. *Genetics* **183,** 639–650 (2009).

18. Zeldovich, K. B., Chen, P. & Shakhnovich, E. I. Protein stability imposes limits on organism complexity and speed of molecular evolution. *Proceedings of the National Academy of Sciences* **104,** 16152–16157 (2007).

19. Goldstein, R. A. The evolution and evolutionary consequences of marginal thermostability in proteins. *Proteins: Structure, Function, and Bioinformatics* **79,** 1396–1407 (2011).

20. Feder, M. E., Bennett, A. F. & Huey, R. B. Evolutionary physiology. *Annual review of ecology and systematics* **31,** 315–341 (2000).

21. Deutsch, C. A. *et al.* Impacts of climate warming on terrestrial ectotherms across latitude. *Proceedings of the National Academy of Sciences* **105,** 6668–6672 (2008).

22. Johansson, F., Orizaola, G. & Nilsson-Örtman, V. Temperate insects with narrow seasonal activity periods can be as vulnerable to climate change as tropical insect species. *Scientific Reports* **10,** 8822 (2020).

23. Baur, J., Zwoinska, M., Koppik, M., Snook, R. R. & Berger, D. Heat stress reveals a fertility debt owing to postcopulatory sexual selection. *Evolution Letters* **8,** 101–113 (2024).

24. Gillooly, J. F., Brown, J. H., West, G. B., Savage, V. M. & Charnov, E. L. Effects of size and temperature on metabolic rate. *science* **293,** 2248–2251 (2001).

25. Dowling, D. K. & Simmons, L. W. Reactive oxygen species as universal constraints in life-history evolution. *Proceedings of the Royal Society B: Biological Sciences* **276,** 1737–1745 (2009).
