# Supplementary material for: Repeatability of evolution and genomic predictions of temperature adaptation in seed beetles
Source: Nat Ecol Evol. 2025 May 16;9(6):1061–74. doi: 10.1038/s41559-025-02716-5 (PMC12148939; doi:10.1038/s41559-025-02716-5)
Supplement: Supplementary file 2 — Reporting Summary [file 41559_2025_2716_MOESM2_ESM.pdf]

nature portfolio

Corresponding author(s): Alexandre Rego

Last updated by author(s): Mar 10, 2025

Reporting Summary

Nature Portfolio wishes to improve the reproducibility of the work that we publish. This form provides structure for consistency and transparency in reporting. For further information on Nature Portfolio policies, see our Editorial Policies and the Editorial Policy Checklist.

Statistics

For all statistical analyses, confirm that the following items are present in the figure legend, table legend, main text, or Methods section.

n/a	Confirmed	
☐	☒	The exact sample size (*n*) for each experimental group/condition, given as a discrete number and unit of measurement
☐	☒	A statement on whether measurements were taken from distinct samples or whether the same sample was measured repeatedly
☐	☒	The statistical test(s) used AND whether they are one- or two-sided<br>*Only common tests should be described solely by name; describe more complex techniques in the Methods section.*
☐	☒	A description of all covariates tested
☐	☒	A description of any assumptions or corrections, such as tests of normality and adjustment for multiple comparisons
☐	☒	A full description of the statistical parameters including central tendency (e.g. means) or other basic estimates (e.g. regression coefficient) AND variation (e.g. standard deviation) or associated estimates of uncertainty (e.g. confidence intervals)
☐	☒	For null hypothesis testing, the test statistic (e.g. *F*, *t*, *r*) with confidence intervals, effect sizes, degrees of freedom and *P* value noted<br>*Give P values as exact values whenever suitable.*
☒	☐	For Bayesian analysis, information on the choice of priors and Markov chain Monte Carlo settings
☒	☐	For hierarchical and complex designs, identification of the appropriate level for tests and full reporting of outcomes
☐	☒	Estimates of effect sizes (e.g. Cohen's *d*, Pearson's *r*), indicating how they were calculated

Our web collection on statistics for biologists contains articles on many of the points above.

Software and code

Policy information about availability of computer code

Data collection	R and Rstudio (v. 2022.07.2 Build 576). Open source.<br>See "Code availability" statement in manuscript.
Data analysis	R and Rstudio (v. 2022.07.2 Build 576). Open source.<br>Code available at Dryad: https://doi.org/10.5061/dryad.bzkh189kd<br>Peer-reviewer link to data and code: http://datadryad.org/stash/share/ioNUncvnoFT2hGKqclYFKKIunBITTfC75W0HMzjmFCo

For manuscripts utilizing custom algorithms or software that are central to the research but not yet described in published literature, software must be made available to editors and reviewers. We strongly encourage code deposition in a community repository (e.g. GitHub). See the Nature Portfolio guidelines for submitting code & software for further information.

Data

Policy information about availability of data

All manuscripts must include a data availability statement. This statement should provide the following information, where applicable:

- Accession codes, unique identifiers, or web links for publicly available datasets
- A description of any restrictions on data availability
- For clinical datasets or third party data, please ensure that the statement adheres to our policy

European Nucleotide Archive acession code: PRJEB86644

Research involving human participants, their data, or biological material

Policy information about studies with human participants or human data. See also policy information about sex, gender (identity/presentation), and sexual orientation and race, ethnicity and racism.

Reporting on sex and gender	N/A
Reporting on race, ethnicity, or other socially relevant groupings	N/A
Population characteristics	N/A
Recruitment	N/A
Ethics oversight	N/A

Note that full information on the approval of the study protocol must also be provided in the manuscript.

Field-specific reporting

Please select the one below that is the best fit for your research. If you are not sure, read the appropriate sections before making your selection.

☐ Life sciences ☐ Behavioural & social sciences ☒ Ecological, evolutionary & environmental sciences

For a reference copy of the document with all sections, see nature.com/documents/nr-reporting-summary-flat.pdf

Ecological, evolutionary & environmental sciences study design

All studies must disclose on these points even when the disclosure is negative.

Study description

12 Experimental evolution lines and their 3 ancestal lines, of the seed beetle Callosobruchus maculatus, were analyzed.
6 lines were evovled at hot (35C) temperature, and 6 lines were evolved at cold (23C) temperature. The ancestors were kept at ancestral lab temperautre (29C). The evolved lines were created from the ancestors, that originally were sampled from Brazil, Yemen and California (USA). Thus experimental evolution was carried out on three different genetic backgrounds, with two biological replicates per evolution regime and and background.

Evolution lines were scored for their life history adaptation by measuring seven traits at generations 45-60 and again for lifetime offspring production at generations 80-120. Ancestors were measured for their traits at generation 120. Genomic sampling was also conducted at generation 60 in evolved lines and at generation 0 in the ancestor.

We quantified repeatability in both phenotypic and genomic evolution by calculating using vector analyses of evolutionary change. We subsequently modeled the predictability of fitness and phenotypic traits from genomic data across and between geographic origins.

Research sample

Callosobruchus macualtus seed beetles (as desribed above). 12 evolved + 3 ancestral lines. Most measurements were done on females, as these are more directly affecting population growth rates.

Sampling strategy

We measured several thousands of beetles to attain relatively accurate estimates of means for all 7 life-history traits of each line. For these traits, our lab has measured them before, we therefore had a good idea of what was needed, even though no direct power analysis was conducted. Having that said, we also were limited practically, so we also pushed on and tried to measure life history traits for as many beetles as possible for the assays of metabolic rate and related traits.

We maximized statistical power of genomic sampling by pooling individuals from a particular line into DNA libraries. We sequenced pools of individuals to sufficient depth to have high accuracy in estimating allele frequencies (>20X coverage).

Data collection

Life-history traits
We quantified thermal adaptation in female life-history by measuring three core traits: lifetime reproductive success (LRS), juvenile development time and adult body mass, and four rate-dependent traits: early fecundity, weight loss, water loss, and mass-specific metabolic rate (ml CO_2/mass/min) over the first 16h of female reproduction. All life-history traits were collected at generations 40 for cold-adapted lines and 60 for hot-adapted lines, in a large common garden experiment including the two assay temperatures corresponding to the experimental evolution treatments (23°C and 35°C). Ancestral lines were scored in the same experimental conditions with the addition of the ancestral 29°C assay temperature, but on a later occasion following ca. 125 generations of experimental evolution. Note that the ancestors had been kept at the ancestral conditions, to which they had already adapted for more than 300 generations prior to the start of experimental evolution. It can therefore be assumed that the measured trait values correspond well with the trait values at the start of experimental evolution. To control for potential differences in the separate experiments on ancestors and evolved lines stemming from unknown sources, we reared an independent laboratory adapted reference population in both experiments. This indicated that differences in rearing had affected the life-history traits scored over the first 16h of reproduction. We therefore standardized the traits scored during respirometry of the three founding ancestors by this estimated amount (adult mass: increased by 6.4%, metabolic rate: reduced by 12%; early fecundity: reduced by 18%; water loss:

reduced by 15%, and weight loss: reduced by 25%) in order not to erroneously assign these differences to evolutionary divergence between ancestors and evolved lines. Note that this was done averaged across the three assay temperatures and geographic origins. Therefore, our approach to provide more accurate measures of evolutionary divergence between evolved lines and ancestors did not affect the estimated temperature-dependence of adaptation or the importance of geographic differences.

Before assays of life-history traits, non-genetic parental effects were removed by moving F0 grandparents of the assayed individuals into a common temperature of 29°C to lay eggs. The emerging beetles in the next (parental) F1 generation were allowed to mate and lay eggs on beans provided ad libitum. Following 48 hours of egg laying, the beans were split and assigned to one of the two (for ancestors, three) assay temperatures. The emerging adult F2 offspring were phenotyped for their life-history (Fig. 4). Newly emerged (0-48 hours old) virgin females were mated to males by placing three males and females together in a petri dish over night at the assay temperature. In the following morning, the three females were weighed for their body mass and then placed together inside a glass vial filled with black eyed beans to be measured for their metabolic rate, water loss and early fecundity at their designated assay temperature. The glass vials were placed in a Sable Systems (Las Vegas, NV, USA) high-throughput respirometry system. Briefly, the respirometry was set up in stop-flow mode, and CO2 production and water-loss was measured for up to 23 vials on a given experimental day. The first vial was left empty and served as a baseline to control for any drift of the gas analysers during each session. Vials were measured over 17 cycles, each of a length of 57.5 minutes. Mean metabolic rate and water loss for each vial was calculated across cycles 2-17, with the readings from the first cycle discarded (as it contains human-produced water and CO2). After respirometry, females were weighed again to record their weight loss and beans with eggs were isolated and counted to record early fecundity. In total we followed 386 triads of females for the evolved lines and another 115 triads from their ancestors.

From the same rearing we measured egg-to-adult development time for two technical replicates per line and assay temperature, each consisting of 40-120 individuals. We calculated a mean development time per technical replicate and used this in analysis. We also collected virgin males and females and placed three males and three females together in a petri dish with ad libitum beans to record lifetime reproductive output (LRS) at each assay temperature. In total we recorded LRS for 258 couple triplets for evolved lines, and another 115 couple triplets for the ancestors. These data were complemented with additional data from both evolved and ancestral lines reared in a common garden design in two consecutive years (corresponding to generation 120/135 for ancestors, 115/130 for hot-adapted lines, and 80/90 for cold-adapted lines). In these rearings, a single male and female were put together in a petri dish with ad libitum host seeds, with otherwise the same conditions. For ancestors we scored 396 couples, and for evolved lines 789 couples, across both experimental years. LRS was analysed per female, hence we divided all offspring counts from female triads by three before analysis.

Timing and spatial scale

Life history traits were scored in April-June 2018 (generations 45-60) for evolved lines, and for ancestors in April-May 2022. Because ancestors and evolved lines were not scored at the same time for these traits, we also included data for lifetime time offspring production measured in a common garden (including both) at generation January-March 2023.

DNA extraction and sequencing was also conducted during the same time that life history traits were measured (2018).

Data exclusions

N/A

Reproducibility

As described above, we measured lifetime offspring production twice to verify that differences remained between evolution regimes and ancestors, which resulted in concordant results between the two samples.

Randomization

We reared populations and always tried to run samples from each line on the same day. This was not possible to do on all days as the lines have evolved differences in development time, and beetles develop at different rates in different temperatures. Hence, while lines were started at the same time in the experiment, they were not finishing at the same time, and for some traits they were measured on different days.

Blinding

For all assays we used ID numbers and not the actual name of the lines. However, since beetles develop predictably from different temperature treatments, it was not possible to blind the observer from this particular aspect of the experimental design.

Did the study involve field work? ☐ Yes ☒ No

Reporting for specific materials, systems and methods

We require information from authors about some types of materials, experimental systems and methods used in many studies. Here, indicate whether each material, system or method listed is relevant to your study. If you are not sure if a list item applies to your research, read the appropriate section before selecting a response.

Materials & experimental systems

n/a	Involved in the study	
☒	☐	Antibodies
☒	☐	Eukaryotic cell lines
☒	☐	Palaeontology and archaeology
☐	☒	Animals and other organisms
☒	☐	Clinical data
☒	☐	Dual use research of concern
☒	☐	Plants

Methods

n/a	Involved in the study	
☒	☐	ChIP-seq
☒	☐	Flow cytometry
☒	☐	MRI-based neuroimaging

Animals and other research organisms

Policy information about studies involving animals; ARRIVE guidelines recommended for reporting animal research, and Sex and Gender in Research

Laboratory animals	Callosobruchus maculatus (strains from Brazil, Yemen, and USA).
Wild animals	N/A
Reporting on sex	Only females were measured.
Field-collected samples	Laboratory stocks which have been maintained >10 years before experiments (according to standard lab conditions - 29C and 50-55% RH)
Ethics oversight	No ethical approval is necessary for insects according to national legislation.

Note that full information on the approval of the study protocol must also be provided in the manuscript.

Plants

Seed stocks	*Report on the source of all seed stocks or other plant material used. If applicable, state the seed stock centre and catalogue number. If plant specimens were collected from the field, describe the collection location, date and sampling procedures.*
Novel plant genotypes	*Describe the methods by which all novel plant genotypes were produced. This includes those generated by transgenic approaches, gene editing, chemical/radiation-based mutagenesis and hybridization. For transgenic lines, describe the transformation method, the number of independent lines analyzed and the generation upon which experiments were performed. For gene-edited lines, describe the editor used, the endogenous sequence targeted for editing, the targeting guide RNA sequence (if applicable) and how the editor was applied.*
Authentication	*Describe any authentication procedures for each seed stock used or novel genotype generated. Describe any experiments used to assess the effect of a mutation and, where applicable, how potential secondary effects (e.g. second site T-DNA insertions, mosiacism, off-target gene editing) were examined.*
